# Genome sequences of *Pseudomonas* sp. strain FFPRI_1 and *Photorhabdus temperata* strain FFPRI_2, isolated from the entomopathogenic nematode, *Heterorhabditis megidis*

**DOI:** 10.1128/mra.00752-24

**Published:** 2024-09-24

**Authors:** Jun Takatsuka, Noritoshi Maehara

**Affiliations:** 1 Forestry and Forest Products Research Institute, Forest Research and Management Organization, Tsukuba, Ibaraki, Japan; The University of Arizona, Tucson, Arizona, USA

**Keywords:** entomopathogenic nematode, symbiont, entomopathogen

## Abstract

The complete genome sequences of two bacteria isolated from an entomopathogenic nematode are reported. The genome sizes of *Pseudomonas* sp. strain FFPRI_1 and *Photorhabdus temperata* strain FFPRI_2 are 7,521,420 bp long with 63% G+C content and 5,483,510 bp long with 43.8% G+C content, respectively.

## ANNOUNCEMENT

Recent studies identified a diverse assembly of bacteria associated with entomopathogenic nematodes (EPNs) (1–3), which is beneficial as the bacteria, their toxins, and secondary metabolites have potential uses in agriculture and clinical applications (1, 4, 5). We isolated an EPN, *Heterorhabditis megidis*, from the soil of a Japanese red pine forest in Iwate Prefecture (6). The EPN (approx. 4,000 individuals), with or without surface sterilization (0.1% benzethonium chloride for 30 min) for the isolation of strains FFPRI_2 and FFPRI_1, respectively, was homogenized using a hand homogenizer (BioMasher II: Nippi), and bacteria were isolated on nutrient agar at 30°C. Strain FFPRI_1 produced a 1–2-mm-diameter circular white colony after 24 h, whereas strain FFPRI_2 produced a circular pale pink colony of the same size after 48 h. Bacterial strains were established by streaking on nutrient agar three times, followed by storage at −80°C. Sequencing the genomes of these two strains provides insight into their potential use, particularly as entomopathogens for insect pest control. Freeze stocks were streaked out, and single colonies were cultured in a Luria broth medium at 30°C for 24 h. Bacterial DNA was extracted using the Gentra Puregene yeast/bacteria kit (Qiagen) for sequencing across all platforms.

For strain FFPRI_1, a pair-end sequence library with a 350-bp insert was constructed using a TruSeq DNA PCR-free kit (Illumina), and 151-base paired-end reads were generated using the NovaSeq 6000 platform (Illumina) at Macrogen Japan Corp. (Tokyo, Japan). A long-read sequencing library was prepared using a Ligation Sequence Kit [Oxford Nanopore Technologies (ONT)] without DNA shearing and size selection and sequenced using the GridION X5 platform with R.9.4.1 flow cell (ONT) at Bioengineering Lab. (Kanagawa, Japan). Guppy (ver. 4.0.11+ f1071ce) was used for base-calling and barcode trimming. For Illumina short reads, adapter-trimming, quality-filtering, and removal of <75 base reads were performed using RabbitQC (7). Nanopore long reads were trimmed for adaptors using Porechop ver. 0.2.4 (https://github.com/rrwick/Porechop). Reads with <1,000 bases and the worst 5% were eliminated using Filtlong ver. 0.2.1 (https://github.com/rrwick/Filtlong). A hybrid assembly of the quality-filtered Illumina 68,122,880 and Nanopore 179,109 reads [N50 of 21,001 estimated using nanoplot ver. 1.42.0 (8)] was performed using Unicycler ver. 0.5.0 (9). Circularity was assessed using Unicycler ver. 0.5.0 and Bantage ver. 0.8.1 (10). For strain FFPRI_2, a sequencing library was prepared and sequenced by the Bioengineering Lab. DNA was sheared to 10–20 kb length using a gTUBE (Covaris). The sequence library was prepared using the SMARTbell Express Template Prep Kit 2.0 (Pacific Biosciences) and sequenced with the Sequel IIe system (Pacific Biosciences). SMART Link v10.1.0.119528 was used to generate 27,034 HiFi reads with an N50 of 9,113 that were then assembled using Flye ver. 2.9 (11). The assembly was circularized using Circlator ver. 1.5.5 (12). Annotation and taxonomic assignments were performed using DFAST (13) and FastANI (14) in DFAST. 16S rRNA gene sequences were subjected to BLAST search against the NCBI database, rRNA_typestrains/16S_ribosomal_RNA. Default parameters were used for all software unless otherwise noted.

The assembly resulted in a circular genome for each bacterium. The genomic features are summarized in Table 1. FastANI and BLAST identified strains FFPRI_1 and FFPRI_2 as *Pseudomonas* sp. and *Photorhabdus temperata*, respectively.

**TABLE 1:** Genome features of the bacterial strains

Bacterial strain	FFPRI_1	FFPRI_2
Genome size (bp)	7,521,420	5,483,510
Genome coverage	1,543	42
Number of coding sequences	6,702	4,624
GC content	63	43.8
Number of rRNA	19	22
Number of tRNA	80	77
Number of CRISPR	0	4
Highest average nucleotide identity against type strains^*a*^	90.92% against *Pseudomonas protegens* CHA0 (Accession: GCF_000397205.1)	98.74% against *Photorhabdus temperata* DSM 14550 (Accession: GCF_025384845.1)
Highest identity of 16S rRNA gene sequence against type strains^*b*^	99.93% against *Pseudomonas protegens* CHA0 (Accession: CP003190)	99.93% against *Photorhabdus temperata* strain XINach (Accession: NR_028871)

^*a*^ FastANI (14) in DFAST (13) was used to estimate the values.

^*b*^ BLAST search was performed against the NCBI database, rRNA_typestrains/16S_ribosomal_RNA.

## Data Availability

The genome sequence of *Pseudomonas* sp. strain FFPRI_1 has been deposited in DDBJ/EMBL/GenBank under accession number AP029168 and is associated with BioProject accession number PRJDB17252. The raw sequence read data are available at the DDBJ Sequence Read Archive (SRA) under accession numbers DRR523862 (Illumina NovaSeq 6000) and DRR523863 (GridION). The genome sequence and associated information for *Photorhabdus temperata* strain FFPRI_2 are available at DDBJ/EMBL/GenBank accession number AP029167, BioProject accession number PRJDB17253, and SRA accession number DRR523859. PCR-amplified 16S rRNA gene sequences are available in DDBJ/EMBL/GenBank under accession numbers LC799816 (*Pseudomonas* sp. strain FFPRI_1) and LC799817 (*Photorhabdus temperata* strain FFPRI_2).
